# Repurposing Clinical Molecule Ebselen to Combat Drug Resistant Pathogens

**DOI:** 10.1371/journal.pone.0133877

**Published:** 2015-07-29

**Authors:** Shankar Thangamani, Waleed Younis, Mohamed N. Seleem

**Affiliations:** Department of Comparative Pathobiology, Purdue University College of Veterinary Medicine, West Lafayette, IN, United States of America; Cornell University, UNITED STATES

## Abstract

Without a doubt, our current antimicrobials are losing the battle in the fight against newly-emerged multidrug-resistant pathogens. There is a pressing, unmet need for novel antimicrobials and novel approaches to develop them; however, it is becoming increasingly difficult and costly to develop new antimicrobials. One strategy to reduce the time and cost associated with antimicrobial innovation is drug repurposing, which is to find new applications outside the scope of the original medical indication of the drug. Ebselen, an organoselenium clinical molecule, possesses potent antimicrobial activity against clinical multidrug-resistant Gram-positive pathogens, including *Staphylococcus*, *Streptococcus*, and *Enterococcus*, but not against Gram-negative pathogens. Moreover, the activity of ebselen against Gram-positive pathogens exceeded those activities determined for vancomycin and linezolid, drugs of choice for treatment of *Enterococcus* and *Staphylococcus* infections. The minimum inhibitory concentrations of ebselen at which 90% of clinical isolates of *Enterococcus* and *Staphylococcus* were inhibited (MIC_90_) were found to be 0.5 and 0.25 mg/L, respectively. Ebselen showed significant clearance of intracellular methicillin-resistant *S*. *aureus* (MRSA) in comparison to vancomycin and linezolid. We demonstrated that ebselen inhibits the bacterial translation process without affecting mitochondrial biogenesis. Additionally, ebselen was found to exhibit excellent activity *in vivo* in a *Caenorhabditis elegans* MRSA-infected whole animal model. Finally, ebselen showed synergistic activities with conventional antimicrobials against MRSA. Taken together, our results demonstrate that ebselen, with its potent antimicrobial activity and safety profiles, can be potentially used to treat multidrug resistant Gram-positive bacterial infections alone or in combination with other antibiotics and should be further clinically evaluated.

## Introduction

Infections caused by Gram-positive drug-resistant pathogens are a leading cause of mortality. Three species—methicillin-resistant *Staphylococcus aureus* (MRSA), *Streptococcus pneumoniae* and vancomycin-resistant enterococcus (VRE)—are responsible annually for at least 84% of the antibiotic-resistant bacteria mortality in the United States alone. Further exacerbating the issue of bacterial resistance is the slow rate of the development and approval of new antimicrobials. For almost 80 years, antimicrobials have been crucial allies in the treatment of bacterial infections caused by these pathogens. However, multidrug resistant strains have recently emerged that are resistant to almost all antimicrobials once deemed effective, including fluoroquinolones, macrolides, and β-lactams [1]. Collectively, this points to an urgent need for the discovery of new antimicrobials and novel strategies to develop them. One novel strategy that warrants more attention as a unique method for development of new antimicrobials is drug repurposing [2]. Our recent attempt to identify non-antibiotic drugs with potent antimicrobial activity, within an applicable clinical range, identified organoselenium compound ebselen (EB) as having potent antibacterial activities against Gram-positive pathogens [3, 4]. EB is considered a clinically safe molecule but without proven use yet [5]. It has anti-oxidative, anti-inflammatory, and anti-atherosclerotic properties [6]. Additionally, EB has been shown to exhibit antimicrobial activity *in vitro* and *in vivo* [3, 4, 7–9]. EB exhibited antimicrobial activity by inhibition of thioredoxin reducatse (TrxR) enzyme of *Escherichia coli* and H^+^-ATPase function and proton-translocation function in yeast [7, 8, 10]. However, the antibacterial mechanism of action of EB against Gram-positive bacteria remains unidentified [8].

The potent antimicrobial activity of EB against Gram-positive pathogens motivated us to further investigate the therapeutic applications of EB. The aims of the present study are to investigate the antibacterial activity of EB against Gram-positive clinical pathogens, including MRSA and VRE *in vitro*, to identify antibacterial mechanism of action, to analyze the ability of EB to clear MRSA intracellular infection, to evaluate antibacterial efficacy in MRSA-infected *Caenorhabditis elegans* whole animal models, to evaluate the effect on mitochondrial biogenesis and toxicity in *C*. *elegans*, and to assess whether EB is capable of working synergistically with conventional antibiotics against MRSA in *in vitro* and in infected cell cultures. This study provided valuable insights into potential therapeutic applications of EB for use as antimicrobial agents for the treatment of multidrug-resistant Gram-positive infections.

## Materials and Methods

### Bacterial strains and reagents

Bacterial strains employed in this study are presented in Table 1. Mannitol salt agar (MSA) was purchased from Hardy Diagnostics (Santa Maria, CA). Muller-Hinton broth (MHB) was purchased from Sigma-Aldrich (St. Louis, MO). Trypticase soy broth (TSB) and Trypticase soy agar (TSA) were purchased from Becton, Dickinson (Cockeysville, MD). EB was purchased from (Adipogen corp, San Diego), vancomycin hydrochloride (Gold Biotechnology, St. Louis, MO), linezolid (Selleck Chemicals, Houston, TX), clindamycin (TCI chemicals, Portland, OR), erythromycin, rifampicin, ampicillin, gentamicin, chloramphenicol and fetal bovine serum (FBS) were purchased from Sigma-Aldrich (St. Louis, MO). DMEM media were purchased from Life technologies and MTS reagent (Promega, Madison, WI, USA).

**Table 1:** The MIC and MBC of EB against Gram-positive and Gram-negative bacteria.

Bacterial Strains	Strain ID	Source	Phenotypic Characteristics	MIC/MBC (μg/ml)
*Enterococcus* spp	*E*. *faecalis* ATCC49533	Blood, Wisconsin	Resistant to streptomycin	0.25/8
	*E*. *faecalis* ATCC7080	Meat involved in food poisoning, New York	-	0.25/8
	*E*. *faecalis* ATCC49532	Blood, Wisconsin	Resistant to gentamicin	0.25/8
	*E*. *faecalis* ATCC14506	Quality control strain	-	0.5/8
	*E*. *faecalis* ATCC 51229 (VRE)	Peritoneal fluid, St. Louis, MO	Resistant to vancomycin. Sensitive to teichoplanin	0.5/0.5
	*E*. *faecalis* SF24397	Urine, Michigan	Resistance to erythromycin (ermB+) and gentamicin	0.125/4
	*E*. *faecalis* SF24413 (VRE)	Urine, Michigan	Resistant to erythromycin, gentamicin and vancomycin	0.125/4
	*E*. *faecalis* SF28073 (VRE)	Urine, Michigan	Resistant to erythromycin, gentamicin and vancomycin	0.0625/8
	*E*. *faecalis* HH22	Urine, Texas	Resistance to penicillin, erythromycin, tetracycline and high levels of aminoglycosides	0.125/4
	*E*. *faecalis* MMH594	Blood, Wisconsin	Resistance to erythromycin and gentamicin	0.125/4
	*E*. *faecalis* SV587 (VRE)	Urine Missouri	Resistance to vancomycin	0.125/8
	*E*. *faecium* E1162	Blood, France	Resistance to ampicillin.	0.25/16
	*E*. *faecium* E0120 (VRE)	Ascites fluid, Netherlands	Resistant to gentamicin and vancomycin	0.5/32
	*E*. *faecium* ERV102 (VRE)	Oral sputum, Colombia	Resistant to ampicillin and vancomycin, and displays high levels of resistance to streptomycin	0.5/16
	*E*. *faecium* ATCC6569	Human feces	-	1/32
	*E*. *faecium* ATCC 700221 (VRE)	Human feces, Connecticut	Resistant to vancomycin and teicoplanin	0.5/1
*Staphylococcus* spp	MSSA (NRS 72)	United Kingdom	Resistant to penicillin	0.25/0.5
	MRSA (NRS 384)	United States (Mississippi)	Resistant to erythromycin, methicillin, and tetracycline	0.125/0.125
	MRSA (NRS119)	United States (Massachusetts)	Resistant to linezolid	0.125/0.25
	MRSA (NRS 123)	United States	Resistant to methicillin; susceptible to nonbeta-lactam antibiotics	0.25/0.5
	MRSA (NRS194)	United States (North Dakota)	Resistant to methicillin	0.25/1
	MRSA (NRS108)	France	Resistant to gentamicin	0.25/0.25
	MRSA (NRS70)	Japan	Resistant to clindamycin, erythromycin and spectinomycin	0.25/0.25
	VISA (NRS 1)	Japan	Resistant to aminoglycosides and tetracycline (minocycline)	0.125/0.125
	VISA (NRS 19)	United States (Illinois)	Glycopeptide-intermediate S. aureus	0.25/0.025
	VRSA11a	United States	Resistant to erythromycin and spectinomycin	0.125/0.25
	VRSA11b	United States	Resistant to erythromycin and spectinomycin	0.25/0.25
	VRSA12	United States	Resistant to vancomycin	0.25/0. 5
	VRSA13	United States	Resistant to vancomycin	0.25/0.25
*Streptococcus* spp	*Streptococcus pyogenes* ATCC 12344	-	Quality control strain	0.5/1
	*Streptococcus agalactiae* MNZ938	Human blood	Beta-hemolytic, Serogroup: Group B	0.5/0.5
	*Streptococcus agalactiae* MNZ 933	Human blood	Beta-hemolytic, Serogroup: Group B	0.5/0.5
	*Streptococcus agalactiae* MNZ 929	Human blood	Beta-hemolytic, Serogroup: Group B	0.5/0.5
Gram-negative bacteria	*Acinetobacter baumannii* ATCC BAA1605	MDR strain isolated from the sputum of a Canadian soldier	Resistant to ceftazidime, gentamicin, ticarcillin, piperacillin, aztreonam, cefepime, ciprofloxacin, imipenem and meropemem	16/ND
	*E*. *coli* O157:H7 ATCC 700728	Quality control strain	-	32/ND
	*Salmonella Typhimurium* ATCC 700720	Isolated from a natural source	-	32/ND
	*Klebsiella pneumonia* ATCC BAA 2146	Human urine	Clinical isolate New Delhi Metallo- β-Lactamase (NDM-1) positive	64/ND
	*Pseudomonas aeruginosa* ATCC 9721	Quality control strain	-	>256/ND

VRE: vancomycin-resistant Enterococcus; MSSA: methicillin-sensitive *S*. *aureus;* MRSA: methicillin-resistant *S*. *aureus;* VISA: vancomycin-intermediate *S*. *aureus;* VRSA: vancomycin-resistant *S*. *aureus;* ND: not determined

### In vitro antibacterial assays

Minimum inhibitory concentrations (MICs) were evaluated using micro dilution broth as per the standards of Clinical and Laboratory Standards Institute (CLSI) [11]. MICs of drugs were interpreted as the lowest concentration of the drug which inhibits the growth of bacteria after incubating for at least 16–24 h at 37°C. The minimum bactericidal concentration (MBC) was determined by sub-culturing 10 μl from the wells were no growth was observed onto TSA plates. The plates were incubated for 24 h before the MBCs were determined. The MBC was categorized as the concentration where ⩾99.9% reduction in bacterial cell count was observed [1].

### Intracellular infection assay

J774A.1 murine macrophage-like cells were seeded at a density of 20,000 cells per well in 96-well tissue culture plates. Cells were infected with MRSA USA300 (NRS 384–0114; ST-8) for 30 min at a 1:100 multiplicity of infection (MOI). Then the cells were washed three times with DMEM medium containing 10 IU lysostaphin to kill the extracellular bacteria [12]. Drugs (vancomycin, linezolid and EB) were added at a concentration of 1 μg/ml to the DMEM medium supplemented with 10% FBS and 4 IU lysostaphin. After 24 h incubation, the cells were washed three times with phosphate buffered saline (PBS) and lysed with 0.1% Triton X-100. Lysates were diluted and plated on TSA plates and MRSA colony forming units (CFU) were counted.

### Toxicity assay

The toxicity assays were performed in cell culture and *C*. *elegans*. (a) Cell culture: J774A.1 murine macrophage-like cells at a density of 20,000 cells per well were seeded and allowed to adhere in a 96-well tissue culture plate in DMEM media containing 10% FBS overnight. EB at various concentrations ranging from 0 to 256 μg/ml were added to the cells in DMEM media with FBS. After 24 h incubation with the drug, cells were washed with PBS and the MTS assay reagent,3-(4,5-dimethylthiazol-2-yl)-5-(3-carboxymethoxyphenyl)-2-(4-sulfophenyl)-2H tetrazolium) in DMEM medium was added and incubated for 4 h at 37°C. Absorbance was measured at 490 nm using ELISA microplate reader (Molecular Devices, Sunnyvale, CA, USA). Cell viability after treatment with EB was expressed as a percentage of the control, DMSO. (b) *C*. *elegans*: Temperature-sensitive *C*. *elegans* AU37 (sek-1; glp-4) strain (glp-4(bn2) was used for toxicity studies and the worms were synchronized as described before [13]. Synchronized L4-stage worms were re-suspended in buffer containing 50% M9 buffer and 50% TSB. Then 100 μl of the buffer containing approximately 15–20 worms were deposited in each well in 96-well plates and EB (4 and 8 μg/ml) and vancomycin (8 μg/ml) were added. Worms were counted daily for three days and the percent of live worms was calculated in each group. At least triplicate wells were used for each treatment

### Cell-free bacterial and mammalian transcription/translation assay

The cell-free bacterial translation and mammalian translation assays were performed by the commercially available *Escherichia coli* S30 System and Rabbit Reticulocyte Lysate System (Promega), respectively. The assays were performed as described by the manufacturer, in conjunction with appropriate positive control (chloramphenicol) and negative control (ampicillin) antibiotics. In bacterial translation assay, the reaction mixtures were incubated at 37°C for 1 h. Mammalian translation assay reaction mixtures were incubated at 30°C for 1 h. Luciferase assay reagent was added to the reaction and the intensity of the luminescence was measured by luminescence microplate reader (FLx800 BioTek Instruments, Inc. Winooski, Vermont) according to the manufacturer’s instructions. Average luciferase readout of protein production from two replicates from two independent experiments was calculated.

### Mitochondrial biogenesis assay

The mitobiogenesis assay was done using In-Cell ELISA Kit (MitoSciences Inc., Eugene, OR) as per the manufactures instruction [14]. Briefly, J774A.1 cells were seeded (40,000 cells per well) in 96-well plates for overnight. EB and control antimicrobials (chloramphenicol and ampicillin) were added to the cells and the cells were allowed to grow for approximately 3 days with the drugs. Media were removed and cells were washed with PBS, then fixed with 4% paraformaldehyde. After fixing, cells were washed with PBS and permeabilization and blocking processes were done according to the manufacturer’s instructions. Primary antibodies to detect the levels of two proteins (subunit I of Complex IV (COX-I), which is mitochondrial DNA (mtDNA)-encoded, and the 70 kDa subunit of Complex II (SDH-A), which is nuclear DNA (nDNA)-encoded were added and incubated for overnight at 4°C. After incubation, cells were washed with PBS and secondary antibodies were added and incubated at room temperature for 1 h. The expression of SDH-A and COX-1 were measured after washing and development at 405 nm and 600 nm wavelength, respectively. The ratio between COX-I and SDH-A was calculated and the percent of inhibition of mitochondrial biogenesis was measured.

### Efficacy of EB in infected animal model (*C*. *elegans*)

L4-stage worms of *C*. *elegans* AU37 (sek-1; glp-4) strain (glp-4(bn2) were used to test the antimicrobial efficacy of EB as described before [13]. Briefly, worms were infected with MRSA USA300 (NRS 384–0114; ST-8) in nematode growth media agar plate for 8 h at room temperature. After 8 h of infection, worms were collected and washed with M9 buffer four times before incubation with the drugs. Worms were transferred to 96-well plates (20 worms per well) and the drugs (EB and vancomycin) were added to the wells in triplicates to achieve a final concentration of either 4 or 8 μg/ml. After 24 h incubation with the drugs, worms were transferred to 2-ml centrifuge tubes, washed four times with PBS and 100 mg 1.0-mm silicon carbide particles (Biospec Products, Bartlesville, OK) were added to each tube. The tubes were vortexed for one minute at maximum speed to disrupt the worms without affecting bacterial survival [13]. The resulting suspension was diluted and plated onto MSA plates to count the MRSA CFU. The total CFU obtained from each well was divided by the number of worms in respective wells and the results were expressed as percent of bacterial reduction per worm.

### Synergistic activities of EB with conventional antibiotics in vitro and in cell culture

(a) *In vitro synergistic assay*: The synergistic activities of EB with conventional antibiotics were evaluated using the Bliss Independence Model as described before [4, 15]. Briefly, the optical density of the bacteria grown in the presence of antibiotics and EB (*f*_*AB*_), antibiotics alone (*f*_*A0*_), EB alone (*f*_*0B*_) and in the absence of drugs (*f*_*00*_) were measured and a degree of synergy (S) was calculated using the formula: *S = (f*_*A0*_ */f*_*00*_ *)(f*_*0B*_ */f*_*00*_ *)-(f*_*AB*_ */f*_*00*_ *)*. Positive and negative values represent the degree of synergism and antagonism, respectively. *(b) Intracellular synergistic assay in J774A*.*1 cells*: J774A.1 cells were seeded and infected as described before under intracellular infection assay. EB at concentration of 0.5 μg/ml was added to infected cells alone or in in combination with control antibiotics such as linezolid (4 μg/ml), clindamycin (1 μg/ml), vancomycin (4 μg/ml), chloramphenicol (4 μg/ml), erythromycin (8 μg/ml), rifampicin (0.5 μg/ml) and gentamicin (1 μg/ml). Untreated cells, and cells treated with antibiotics alone were used as a control. After 24 h incubation, the cells were lysed and intracellular MRSA CFU were determined as described above. Percent bacterial reduction was calculated in relative to the untreated groups. Combination therapy was compared with single antibiotic therapy treatment groups.

### Statistical analyses

Statistical analyses were done using Graph Pad Prism 6.0 (GraphPad Software, La Jolla, CA). *P* values were calculated by the one-tailed Student *t* test. *P* values of ˂ 0.05 were considered as significant.

## Results and Discussion

### In vitro antibacterial assays

In an attempt to repurpose approved drugs as antimicrobial agents, we investigated the antimicrobial activity of EB against various multidrug-resistant clinical isolates of Gram-positive and Gram-negative pathogens (Table 1). EB exhibited potent bactericidal activity, in a nanogram range, against all tested Gram-positive strains regardless of their resistance phenotype. EB showed potent activity against clinical isolates of *Enterococcus faecalis* and *Enterococcus faecium* with MIC_90_ of 0.5 μg/ml (see Table 1). EB also showed potent activity against vancomycin-resistant strains of *Enterococcus* (VRE). Next, we tested the activity of EB against the clinical isolates of multidrug-resistant *S*. *aureus*. EB showed more potent activity against methicillin-sensitive *S*. *aureus*, MRSA, vancomycin-intermediate *S*. *aureus* (VISA) and vancomycin-resistant *S*. *aureus* (VRSA) strains than VRE with MIC_90_ of 0.25 μg/ml (see Table 1). Finally, EB also showed potent activity against clinical isolates of *Streptococcus pyogenes* and *Streptococcus agalactiae* with MIC of 0.5 μg/ml (see Table 1). On the other hand, EB did not show potent antimicrobial activity (MIC ≥16 μg/ml) against Gram-negative pathogens, including *Pseudomonas aeruginosa*, *Escherichia coli*, *Klebsiella pneumonia*, *Salmonella Typhimurium*, *and Acinetobacter baumannii*. The lack of activity of EB against Gram-negative pathogens might be due to its reduced ability to enter the cells due to outer membrane barrier or the efflux pump rather than lack of target of EB inside Gram-negative bacteria [16–19].

### Intracellular infection and cell toxicity

**Fig 1:**
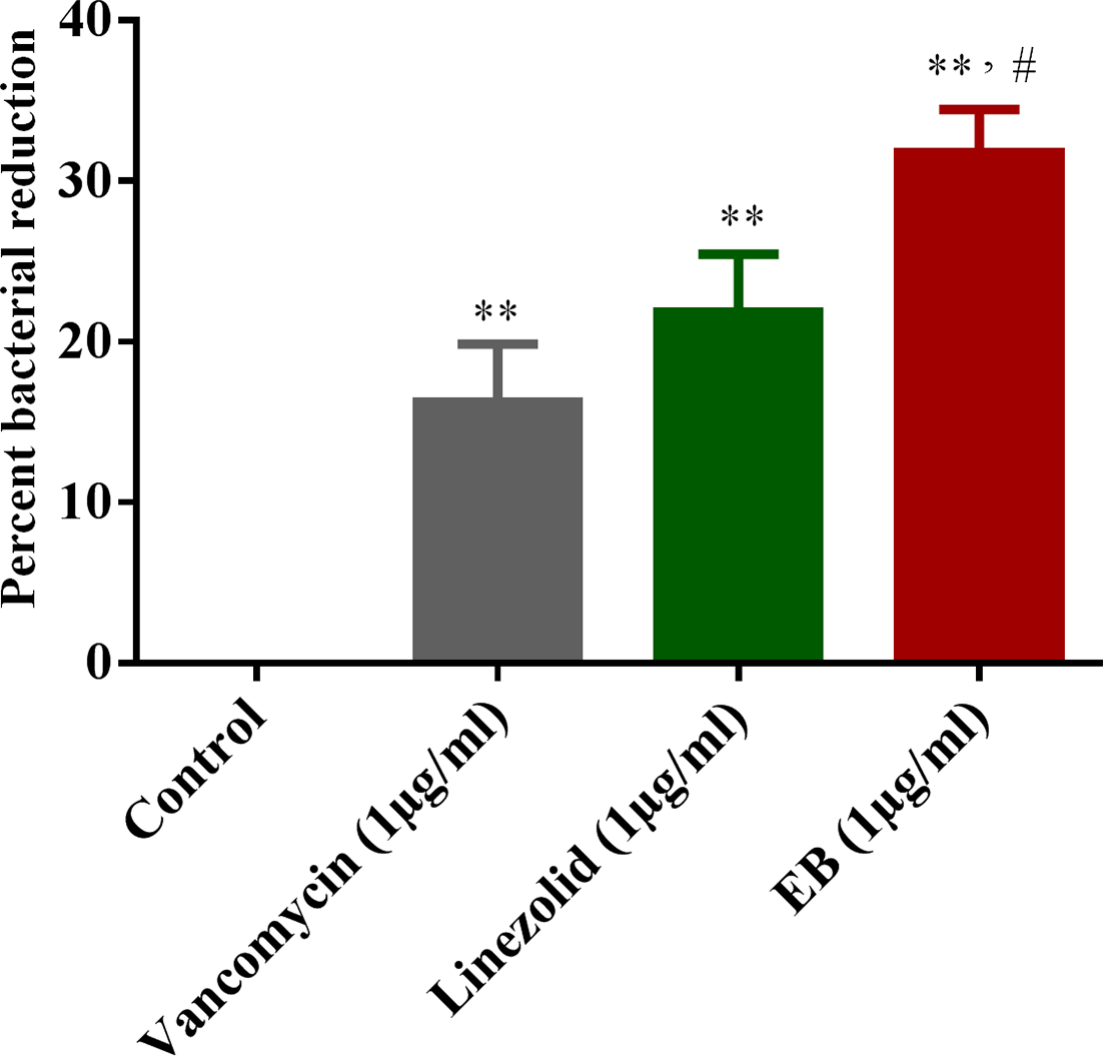
Activity of EB, vancomycin and linezolid against intracellular MRSA USA300 in J774A.1 cells. MRSA infected J774A.1 cells were treated with EB and control antibiotics (vancomycin and linezolid) for 24 h and the percent bacterial reduction was calculated compared to untreated control groups. The results are given as means ± SD (n = 3). *P* values of (**, # ≤ 0.05) are considered as significant. EB was compared to controls (**) and to antibiotics (#).

**Fig 2:**
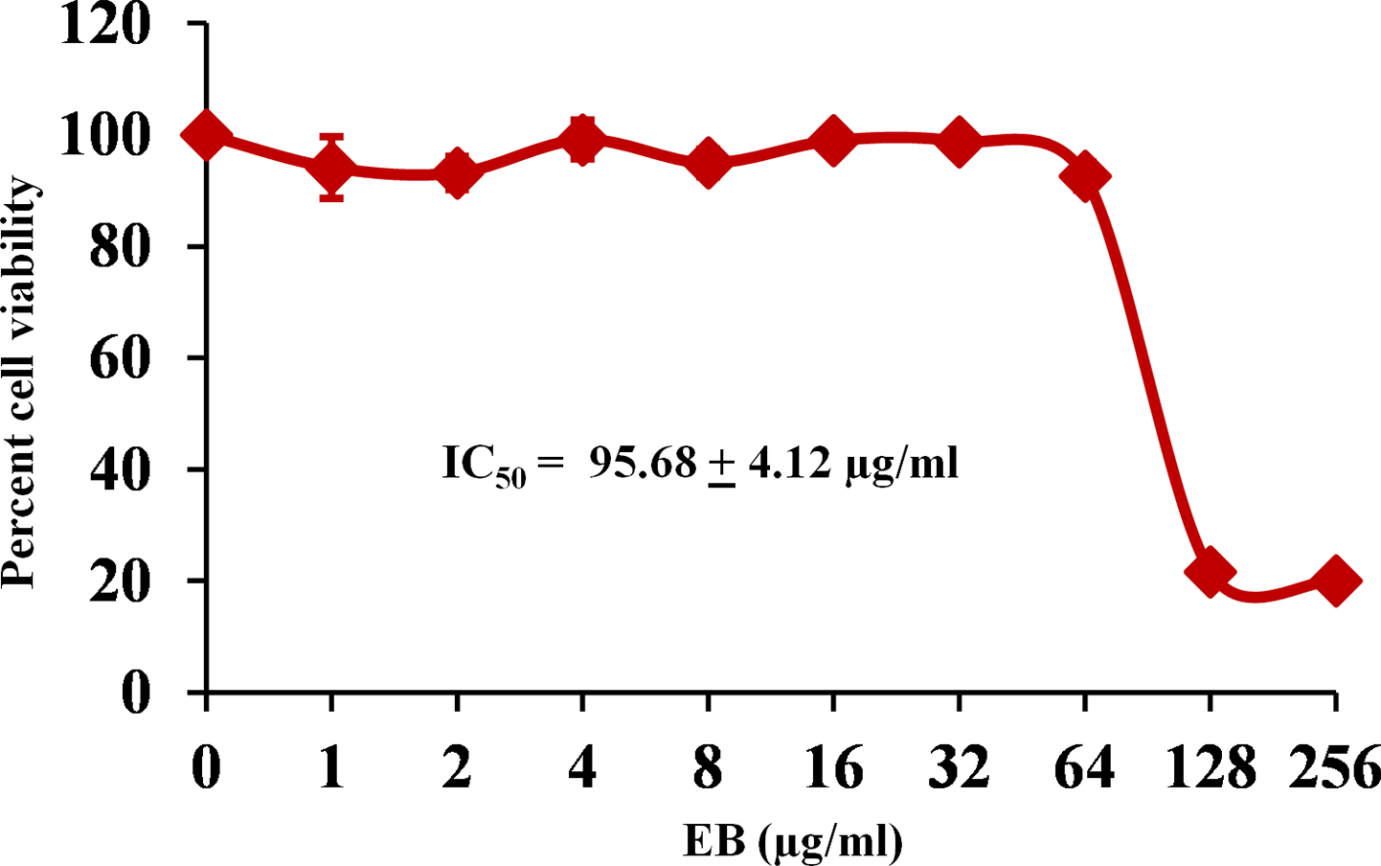
Cytotoxicity assay in murine macrophage-like cells (J774A.1) cells. J774A.1 cells were treated with different concentration of EB ranging from 0 to 256 μg/ml. DMSO was used as a negative control. Cell viability was measured by MTS assay and IC_50_ of EB to cause cytotoxicity in J774A.1 cells was calculated.

Some extracellular pathogens such as *S*. *aureus* are also capable of invading and surviving within the mammalian host cells, leading to persistent chronic infections. Moreover, during the *S*. *aureus* intracellular invasion phase, treatment with antimicrobials is very challenging because most antibiotics do not actively pass through cellular membranes [20–27]. Therefore, clinical failures of drug of choice, such as vancomycin, to cure *S*. *aureus* pneumonia have exceeded 40% and have been attributed mainly to poor intracellular penetration of the drug and consequently to the failure to kill intracellular MRSA in alveolar macrophages [28]. Hence, finding antimicrobials that possess both extra- and intracellular activity would be an optimum strategy to treat such invasive intracellular *S*. *aureus* infections. Therefore, we investigated if EB possesses intracellular anti-staphylococcal activity. As shown in Fig 1, EB at a concentration of 1 μg/ml significantly reduced the intracellular MRSA by 32%. In contrast, the conventional antimicrobials such as vancomycin and linezolid (drugs of choice for treatment of MRSA infections) at the same concentration reduced intracellular MRSA by only 16% and 21%, respectively. EB toxicity was assayed against J774A.1 cells at a concentration ranging from 0 to 256 μg/ml for 24 h. The results shown in Fig 2 indicate that EB does not show toxicity up to 64 μg/ml. The concentration of the EB that causes 50% toxicity (half inhibitory concentration: IC_50_) in J774A.1 cells is 95.68 ± 4.12 μg/ml. This value is more than 380-fold higher than the concentration required to inhibit MRSA. Collectively, these results suggest that EB has great potential for treatment of *S*. *aureus* infections where not only is eradication of extracellular bacteria important, but the killing of intracellular bacteria is also critical [29].

### Cell-free bacterial transcription/translation assay

**Fig 3:**
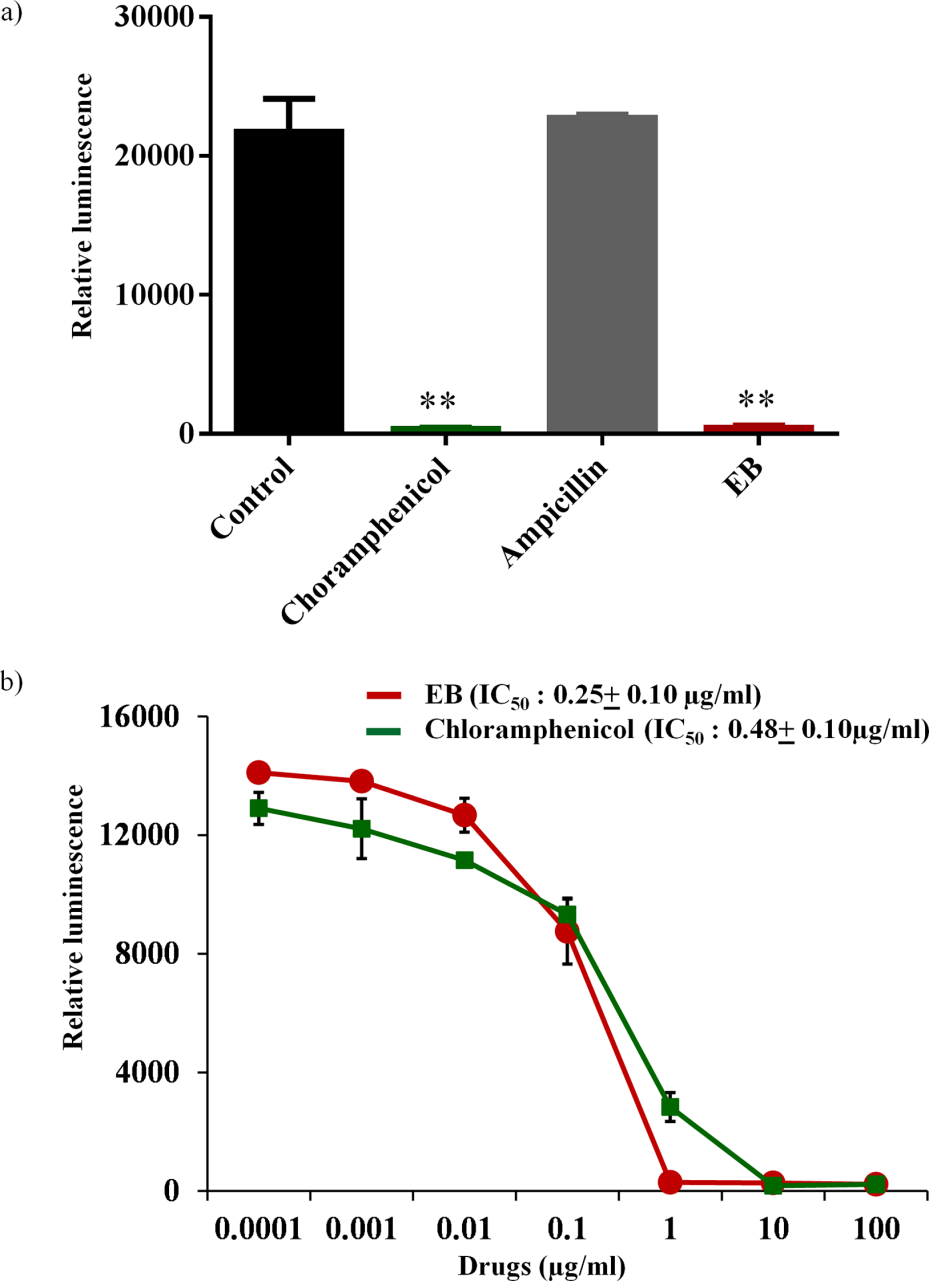
Effects of EB on coupled transcription-translation (TT) in S30 extracts from *E*. *coli*. (a) Average luciferin protein production in the presence of EB, ampicillin and chloramphenicol at the concentration of 2 μg/ml were shown. The results are given as means ± SD (n = 3). (b) Concentration dependent TT-inhibition of EB and chloramphenicol were shown. IC_50_ of the drugs required to inhibit 50% TT-activity were determined. *P* values of (** ≤ 0.05) are considered as significant.

Antimicrobials that target microbial protein synthesis such as oxazolidinones and lincomycins are considered excellent choices for the treatment of toxin-mediated bacterial infections caused by *S*. *aureus*, such as toxic shock syndrome (TSS) and pneumonia [30–33]. In addition to the suppression of *S*. *aureus* toxins such as Panton-Valentine leucocidin (PVL), α-hemolysin (hla), and toxic shock syndrome toxin–1 (TSST-1), these antimicrobials also reduce excessive host-inflammatory responses associated with these toxins [34, 35]. Hence, protein synthesis inhibitors are often preferred in clinical practice for the treatment of toxin-associated staphylococcal infections [30–33]. We tested the effects of EB in our study on bacterial, mammalian and mitochondrial protein-synthesis. For bacterial protein-synthesis inhibition, we used *E*. *coli* cellular extracts in a transcription and translation assay that monitors protein production via luciferase readout. Unlike the antibiotic ampicillin that inhibits cell wall synthesis, EB strongly inhibited bacterial transcription/translation process similar to chloramphenicol antibiotic that inhibits protein synthesis (Fig 3A). EB inhibited bacterial protein synthesis in the cell-free transcription-translation, exhibiting IC_50_ of 0.25±0.10 μg/ml which is comparable to IC_50_ of chloramphenicol antibiotic 0.48 ± 0.10 μg/ml (Fig 3B). These results indicate that EB acts by a favorable mechanism of action and inhibits bacterial protein synthesis and, most likely, toxin production. However, inhibition of bacterial protein synthesis does not exclude other possible mechanism of action of EB.

### Cell-free mammalian transcription/translation assay and mitochondrial biogenesis

**Fig 4:**
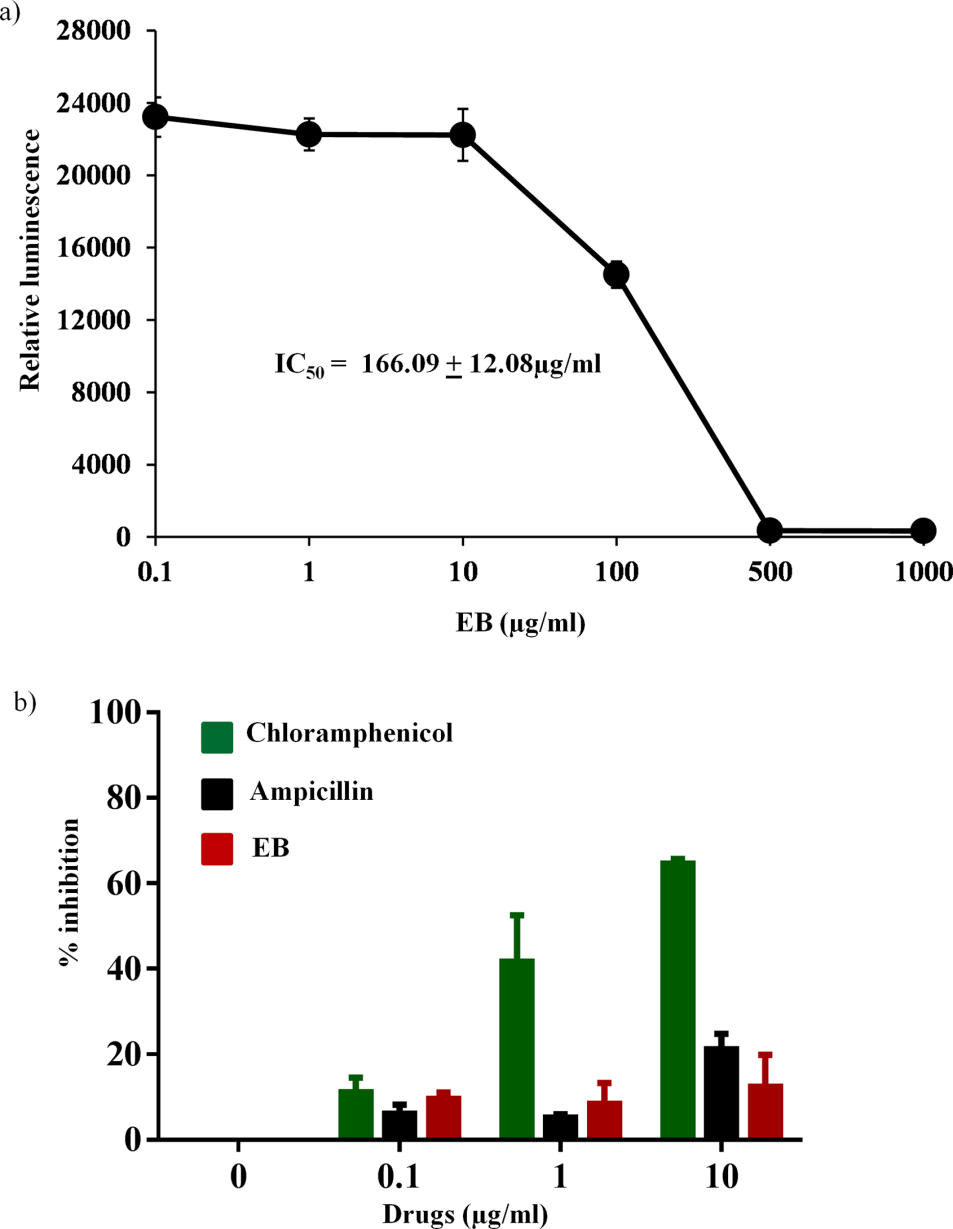
Effects of EB on mammalian protein synthesis. (a) Concentration dependent inhibition of protein synthesis were determined using rabbit reticulocyte lysate extract system. IC_50_ of the EB required to inhibit 50% translational activity were determined. (b) Effect of EB, chloramphenicol and ampicillin on mitobiogenesis. J774A.1 cell In cell- ELISA was carried out in the presence and absence of these drugs, and the levels of mitochondrial (mt)-DNA encoded protein (COX-I) and nuclear-DNA encoded protein (SDH-A) were quantified. Ratio of COX-I and SDH-A were calculated and the results were shown as percent inhibition of mitochondrial biogenesis.

Due to concern about the possible mitochondrial toxicities associated with many antibacterial protein synthesis inhibitors such as linezolid and chloramphenicol [36–42], we tested the effect of EB on the inhibition of eukaryotic transcription/translation process using the rabbit reticulocyte lysate system with the cellular components necessary for mammalian protein synthesis [43, 44]. As shown in Fig 4A, EB showed high safety profile with IC_50_ of mammalian protein synthesis of 166.09 ± 12.08 μg/ml. This value is more than 660-fold higher than the concentration required to inhibit protein synthesis in bacteria. However, in order to test the effect of EB more specifically on mitochondrial biogenesis and to confirm the above *in vitro* results obtained from rabbit reticulocyte lysate system, we measured the effect of EB on mitochondrial protein synthesis directly within the mammalian cells. In-cell ELISA was performed in J774A.1 cells treated with EB and chloramphenicol for three days to detect the levels of mtDNA-encoded COX-I and nDNA-encoded SDH-A proteins. Results shown in Fig 4B indicate that EB had no significant inhibition (less than 10%) of mitobiogenesis, similar to the effect of ampicillin, which does not interfere with mitochondrial protein synthesis process. At the same time, chloramphenicol had more than 60% inhibition of mitochondrial protein synthesis. These results provide valuable information about EB’s safety profile and the lack of interference with mammalian protein synthesis and mitobiogenesis.

### Efficacy of EB in infected animal model (*C*. *elegans*)

**Fig 5:**
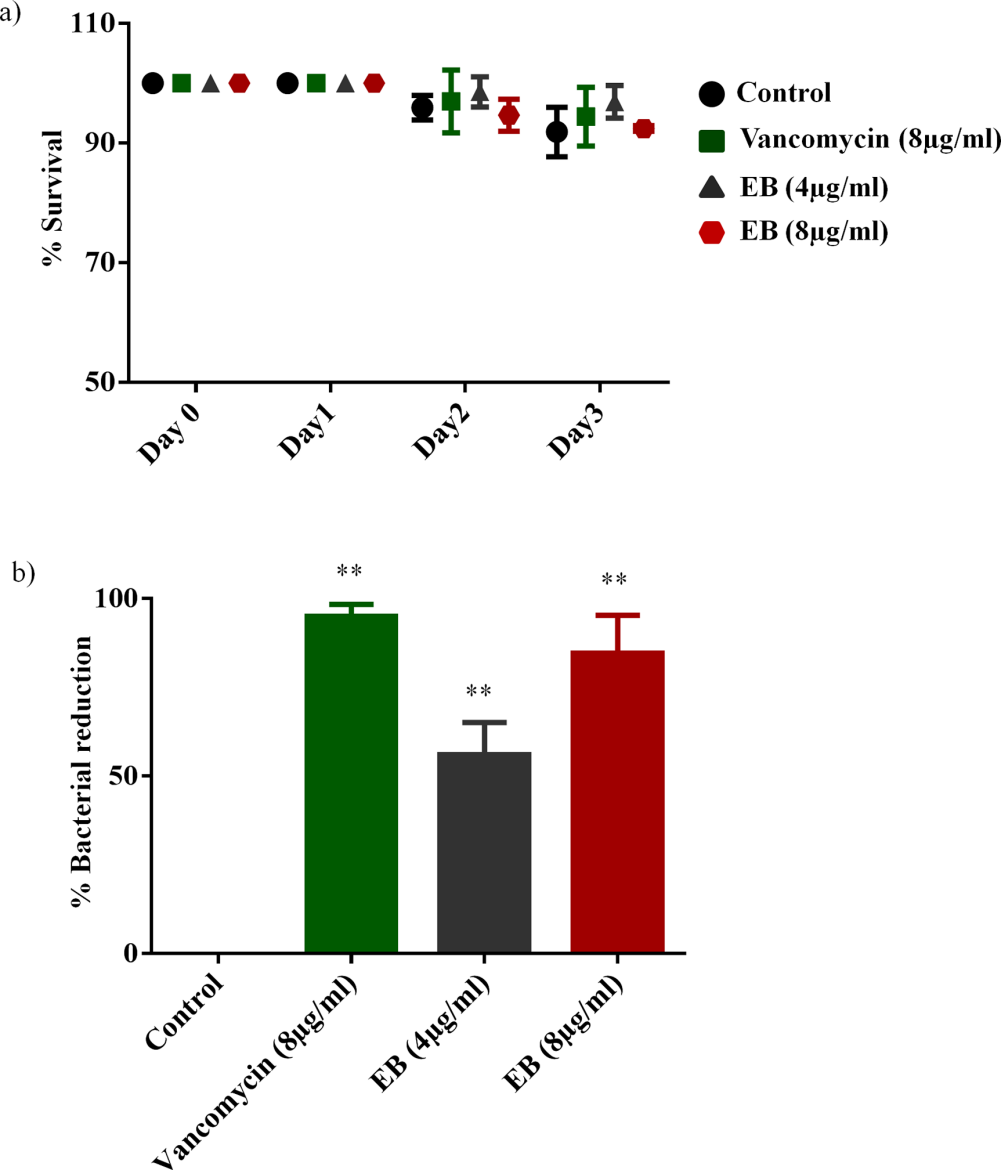
Evaluation of toxicity and antimicrobial efficacy of EB in *C*. *elegans* model. (a) *C*. *elegans* strain glp-4; sek-1 (L4-stage) were grown for three days in the presence of EB (4μg and 8 μg/ml) and vancomycin (8 μg/ml). Live worms were counted and the results were expressed as percent live worms in relative to the untreated control groups. (b) MRSA USA300 infected L4-stage worms were treated with EB (4μg and 8 μg/ml) and vancomycin (8 μg/ml) for 24 h. Worms were lysed and the CFU were counted and the percent bacterial reduction per worm in treated groups were calculated in relative to the untreated control groups. *P* values of (** ≤ 0.05) are considered as significant.

To investigate if the potent *in vitro* antimicrobial activity of EB translates to antimicrobial efficacy *in vivo*, we tested antimicrobial efficacy of EB in an infected *C*. *elegans* whole animal model. A whole animal model, such as *C*. *elegans*, represents a great platform for drug discovery and enables simultaneous assessment of efficacy and toxicity of the tested drugs. Additionally, using a *C*. *elegans* model reduces the associated cost of drug discovery and lowers the burden for extensive animal testing [13, 45]. Prior to testing the efficacy of treatment with EB in infected *C*. *elegans*, we tested toxicity of EB in non-infected *C*. *elegans*. As shown in Fig 5A, treatment of *C*. *elegans* with EB at 4 and 8 μg/ml for three days did not show any significant toxicity, similar to control groups. With no observable toxicity noticed in EB treated groups at a concentration of 4 and 8 μg/ml, we moved forward with an *in vivo* infection model using *C*. *elegans* infected with MRSA. As seen in Fig 5B, treatment with EB had a significant reduction in bacterial load when compared to untreated groups. EB at a concentration of 4 and 8 μg/ml significantly reduced the mean bacterial count by 56% and 85%, respectively. Moreover, treatment with EB at a concentration of 8 μg/ml showed comparable effect to treatment with the drug of choice vancomycin in reducing MRSA burden in infected *C*. *elegans*. Taken together, these results show that EB exhibits potent *in vivo* antistapylococcal efficacy in MRSA-infected *C*. *elegans*.

### Synergistic activities of EB with conventional antibiotics in vitro and in cell culture

**Fig 6:**
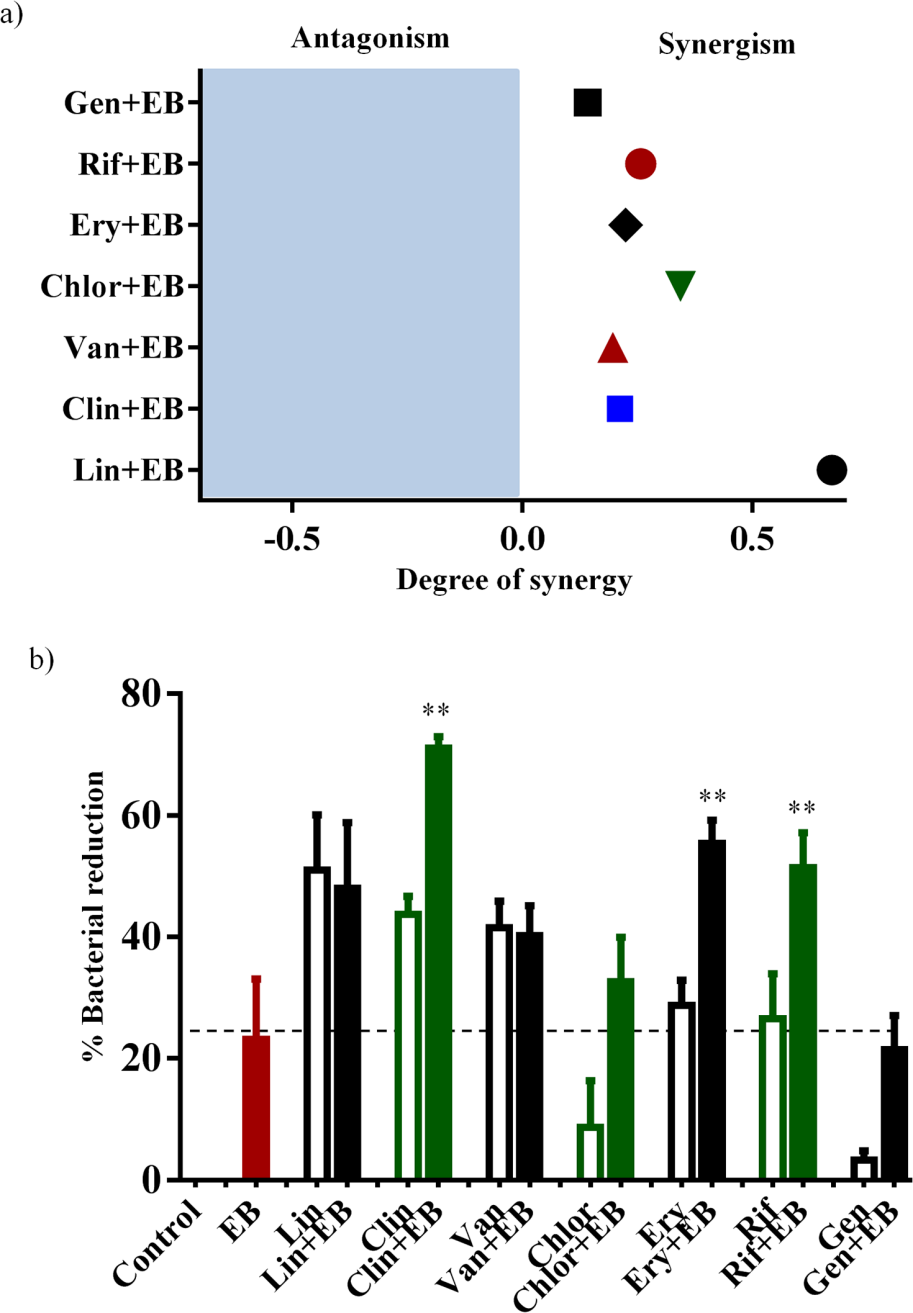
Synergistic activities of EB with conventional antibiotics in vitro and in cell culture. (a) The Bliss Model for Synergy confirms the in vitro synergism with conventional antimicrobials (gentamicin, rifampicin, erythromycin, chloramphenicol, vancomycin, clindamycin and linezolid) against MRSA USA300. Degree of synergy was calculated in the presence of EB (0.0312 μg/ml) in combination with sub-inhibitory concentrations of conventional antimicrobials. (b) Synergistic activity of EB with conventional antimicrobials in infected cell culture. Efficacy of EB (0.5μg/ml) in combination with linezolid (4μg/ml), clindamycin (1μg/ml), vancomycin (4μg/ml), chloramphenicol (4μg/ml), erythromycin (8μg/ml), rifampicin (0.5μg/ml) and gentamicin (1μg/ml) in clearing intracellular MRSA USA300 was determined in J774A.1 cells. Percent bacterial reduction was calculated in relative to the untreated groups. The results are given as means ± SD (n = 3). Combination therapy was compared to monotherapy and the *P* values of (**, ≤ 0.05) are considered as significant.

After confirming that EB has a potential use as an antibacterial agent for the treatment of infections caused by multidrug resistant pathogens, it was important to explore the synergistic relationship of EB with conventional antibiotics *in vitro* and in cell culture. With the rapid emergence of multidrug-resistant strains of *S*. *aureus*, monotherapy with single antibiotic has become less effective [46, 47]. Therefore, alternative strategies such as combinational therapy have been used in the healthcare setting to improve the morbidity associated with MRSA infections and to reduce the likelihood of emergence of resistant strains [1, 46, 48, 49]. To ascertain whether EB has the potential to be combined *in vitro* and in cell culture with conventional antimicrobials such as linezolid, clindamycin, vancomycin, chloramphenicol, erythromycin, rifampicin, and gentamicin against MRSA USA300, we used the *in vitro* Bliss independence model of synergism and infected cell culture assay [15]. *In vitro* results from the Bliss independence model of synergism are presented in Fig 6A. EB was found to exhibit a synergistic relationship with all tested conventional antimicrobials *in vitro* against MRSA USA300. Results of synergistic relationship of EB with conventional antimicrobials in infected cell culture against intracellular MRSA USA300 are presented in Fig 6B. Conventional antimicrobials (clindamycin, erythromycin, and rifampicin) showed synergistic activity when combined with EB and significantly reduced intracellular MRSA when compared to monotherapy. However, EB did not show synergistic activity with linezolid, vancomycin, chloramphenicol, or gentamicin in clearing intracellular MRSA. Identifying antibiotics that can be synergistically paired with EB can potentially prolong the clinical utility of these antibiotics and reduce the likelihood of emergence of resistant strains.

In conclusion, we have successfully explored the potential applications of EB *in vitro*, in cell culture, and *in vivo* to combat multidrug-resistant Gram-positive pathogens, especially MRSA. We demonstrated that EB inhibits the bacterial translation process without affecting mitochondrial biogenesis. Additionally, we demonstrated the efficacy of EB *in vivo* in a *C*. *elegans* MRSA-infected model. Finally, we identified potential antibiotics that can be synergistically combined with EB to prolong the clinical utility of these antibiotics and reduce the likelihood of the emergence of resistant strains. Taken together, our study results demonstrate that EB, with its potent antimicrobial activity and safety profiles, might be a potential candidate drug for systemic and (or) topical applications to treat multidrug resistant Gram-positive bacterial infections alone or in combination with other antibiotics and should therefore be further clinically evaluated.
